# Роль микрохимеризма в развитии эндокринных заболеваний (на примере аутоиммунных заболеваний щитовидной железы и сахарного диабета 1 типа)

**DOI:** 10.14341/probl13636

**Published:** 2026-03-07

**Authors:** М. В. Алавердова, Е. А. Трошина

**Affiliations:** Национальный медицинский исследовательский центр эндокринологии им. академика И.И. ДедоваРоссия; Endocrinology research centreRussian Federation

**Keywords:** микрохимеризм, аутоиммунный тиреоидит, болезнь Грейвса, сахарный диабет 1 типа, беременность, microchimerism, autoimmune thyroiditis, Graves’ disease, type 1 diabetes mellitus, pregnancy

## Abstract

Микрохимеризм - это феномен присутствия в организме генетически чужеродных клеток, представляет значительный интерес для эндокринологии. Формируясь в результате трансплацентарного обмена клетками при беременности (фетальные и материнские микрохимерные клетки) или ятрогенных вмешательств, он может играть важную роль в развитии аутоиммунных эндокринопатий. Наибольшее количество данных накоплено в отношении заболеваний щитовидной железы: фетальные микрохимерные клетки обнаруживаются в 38–83% случаев аутоиммунного тиреоидита и болезни Грейвса, причем их уровень коррелирует с активностью аутоиммунного процесса. Выдвинуты три основные гипотезы их участия в патогенезе: инициация реакции «трансплантат против хозяина» после родов; молекулярная мимикрия с тиреоидными антигенами; пассивное накопление в очагах воспаления. При сахарном диабете 1 типа (СД1) исследования сконцентрированы на материнских микрохимерных клетках, которые выявляются в поджелудочной железе пациентов и могут дифференцироваться в β-клетки, однако их патогенетическая роль остается дискуссионной. Современные методы обнаружения микрохимерных клеток: полимеразная цепная реакция (ПЦР), метод иммунофлюоресцентной гибридизации in situ (FISH — fluorescence in situ hybridization) обладают высокой чувствительностью, но требуют стандартизации. Перспективными направлениями исследований являются изучение влияния человеческих лейкоцитарных антигенов HLA (Human Leukocyte Antigens) — совместимости, долговременной динамики микрохимерных клеток и их потенциального терапевтического применения. Решение этих задач может привести к пересмотру представлений о патогенезе эндокринных заболеваний и разработке новых подходов к их лечению.

## Abstract (en)

Microchimerism — the phenomenon of the presence of genetically foreign cells within the body — holds significant interest for endocrinology. It develops as a result of transplacental cell exchange during pregnancy (fetal and maternal microchimeric cells) or iatrogenic interventions and may play an important role in the development of autoimmune endocrine diseases. The greatest amount of data has been accumulated regarding thyroid diseases: fetal microchimeric cells are detected in 38–83% of cases of autoimmune thyroiditis and Graves’ disease, with their levels correlating with the activity of the autoimmune process. Three main hypotheses have been proposed regarding their involvement in pathogenesis: initiation of a "graft-versus-host" reaction postpartum, molecular mimicry with thyroid antigens, or passive accumulation in inflammatory foci.

In type 1 diabetes mellitus, studies focus on maternal microchimeric cells, which are found in the pancreas of patients and can differentiate into β-cells; however, their pathogenic role remains controversial. Modern methods for detecting microchimeric cells — such as polymerase chain reaction (PCR) and immunofluorescent hybridization in situ (FISH) — are highly sensitive but require standardization. Promising research directions include studying the influence of HLA compatibility, long-term dynamics of microchimeric cells, and their potential therapeutic applications. Addressing these issues could lead to a revision of current understanding of the pathogenesis of endocrine diseases and the development of new treatment approaches.

## Введение

Химера — это организм, который развивается как минимум из четырех родительских клеток и несет в себе более одной ДНК. Формирование химеры происходит различными способами: например, при слиянии двух оплодотворенных яйцеклеток в один эмбрион на ранней стадии развития (тетрагаметный химеризм); при многоплодной беременности, если произошла гибель одного из плодов, его ткани и кровь могут попасть в организм близнеца (двойной химеризм); также химеризм может возникнуть в результате пересадки органов или переливания крови от другого человека (искусственный химеризм).

Микрохимеризм (МХ) — это явление, характеризующееся наличием небольшой популяции клеток с различной генетической информацией внутри организма. Это физиологическое явление, которое обычно встречается у людей и с большой вероятностью встречается у плацентарных млекопитающих в целом. Во время беременности происходит перенос фетальных клеток в ткани матери (фето-материнский микрохимеризм), материнских клеток в ткани плода (материнско-фетальный микрохимеризм) и даже старших братьев, сестер или других эмбрионов, присутствующих в матке, в ткани плода (фето-фетальный микрохимеризм) [1].

Микрохимеризм изучался при некоторых эндокринных заболеваниях. Первые исследования были сосредоточены на фетальных микрохимерных клетках (ФМК) при аутоиммунных тиреоидных патологиях [2][3]. Так, фетальные микрохимерные клетки были обнаружены в щитовидной железе у пациентов с болезнью Грейвса и тиреоидитом Хашимото [4]. Последующие же исследования были посвящены изучению ФМК как при доброкачественных, так и злокачественных узловых заболеваниях щитовидной железы. Наконец, были обнаружены материнские микрохимерные клетки (ММК), которые, как полагают, участвуют в развитии СД1 у потомства [5]. Циркулирующие ФМК в материнской крови были описаны у пациентов с системным [6] и рассеянным склерозом [7], в слюнных железах у больных системной склеродермией [8], в пораженной ткани при локализованной склеродермии [9], в цервикальной ткани у пациентов с раком шейки матки [10], в печени больных первичным билиарным циррозом [11] и в синовиальной ткани больных ревматоидным артритом [12].

## Механизмы развития фетального микрохимеризма

Во время беременности клетки плода проникают через плаценту и попадают в материнский кровоток [13][14]. Мать становится микрохимерной [4][15][16]. После того, как клетки плода поселяются в материнских тканях, например, таких как щитовидная железа, они могут выживать, не разрушаясь, благодаря иммунной адаптации матери во время беременности [17–19]. Это иммунное отклонение во время беременности может сохраняться в течение нескольких месяцев после родов, что позволяет микрохимерным клеткам плода закрепляться и выживать в послеродовой период в организме матери. В норме большая часть фетальных клеток в последующем погибает, тем не менее, есть публикации, где сообщается об их обнаружении спустя много лет [20].

Определенная совместимость HLA (Human Leukocyte Antigens) — между матерью и плодом может иметь последствия для типа или количества плодных клеток, которые сохраняются у матери [21]. Матери и их потомство разделяют один гаплотип HLA и чаще всего различаются по другому гаплотипу, поскольку гены HLA высоко полиморфны. Однако иногда мать и ребенок имеют схожие аллели HLA на своем несовместном гаплотипе HLA. Например, иммуногенетические маркеры восприимчивости HLA DQA1*0501-DQB1*0201 и DQB1*0301, чаще наблюдаемые у пациентов с аутоиммунными заболеваниями щитовидной железы, также чаще встречаются у пациентов пар «мать-ребенок» с фетальным микрохимеризмом [22].

Во время беременности в кровотоке матери обнаруживаются трофобласты плода, ядросодержащие эритроциты, Т- и В-лимфоциты, моноциты, естественные клетки-киллеры (NK) и гемопоэтические клетки-предшественники (клетки CD34 + или CD34 + CD38 +) [23]. Показано, что клетки плода у беременных мышей экспрессируют как маркеры клеток-предшественников, так и маркеры дифференцированных клеток [24]. Чтобы сохраниться после родов, фетальные микрохимерные клетки должны обладать способностью к долгосрочному выживанию в организме матери и, следовательно, должны разделять свойства стволовых клеток, такие как неограниченная способность к самообновлению и пластичность для многолинейной дифференцировки [25]. Эти клетки были названы клетками-предшественниками, связанными с беременностью [17]. Также описан перенос фетальных гемопоэтических клеток-предшественников, фетальных мезенхимальных стволовых клеток или эндотелиальных клеток-предшественников к матери. Они могут представлять собой долгосрочный резервуар стволовых клеток с многолинейным потенциалом [26].

## Лабораторные методы, используемые для определения МХ в организме

МХ могут быть выявлены в периферической крови и тканях. Проще всего микрохимерные клетки выявить у женщин, имевших в анамнезе беременность плодом мужского пола, путем обнаружения в их организме клеток с мужской ДНК. В качестве маркеров служат участки Y-хромосомы — SRY-ген, локализованный на ее коротком плече, и/или DYS14, находящийся в интроне-1 многокопийного гена, кодирующего белок TSPY (Testis-Specific Protein Y-Encoded). Для амплификации этих участков используется полимеразная цепная реакция (ПЦР) [26].

Cirello V. и соавт. в своем исследовании оценивали ФМК с помощью ПЦР в периферической крови, а хромосому Y идентифицировали с помощью флуоресцентной гибридизации in situ в некоторых тканях больных с болезнью Грейвса (БГ). Типирование полиморфизма HLA-G оценивали с помощью ПЦР в реальном времени. В результате ФМК встречались значительно чаще у женщин без заболевания (63,6%), чем у женщин с БГ (33,3%) или тиреоидитом Хашимото (ТХ) (27,8%) (р=0,0004 и р=0,001 соответственно). Количественный анализ подтвердил, что циркулирующая мужская ДНК была более распространена у здоровых женщин, чем у женщин с БГ или ТХ. Микрохимерные клетки были обнаружены в сосудах и фолликулах щитовидной железы. Ни у пациентов с БГ/ТХ, ни у здоровых женщин типирование HLA-G не отличалось между ФМК-положительными и ФМК-отрицательными случаями. Плацентарные факторы были исключены из числа детерминант обнаруженных различий. Сосудистая и тканевая локализация микрохимерных клеток дополнительно подчеркивает способность этих клеток мигрировать в поврежденные ткани [27].

С помощью качественной ПЦР можно выявить факт наличия микрохимеризма [28]. Количественная ПЦР в режиме реального времени позволяет отобразить количество микрохимерных клеток на 100 000 клеток организма-хозяина. Так, Ando T. и соавт. обнаружили интратиреоидный фетальный микрохимеризм с низким количеством мужских клеток у нормальных мышей во время беременности, соответствующий низкому уровню неспецифического накопления [29]. Klintschar et al. [30], изучая феномен фетального микрохимеризма у женщин с тиреоидной патологией, не смогли найти ни одного образца аденомы щитовидной железы с положительными мужскими клетками. Другие сообщили о низком количестве мужских клеток в аденоме щитовидной железы [31][32], аденоматозном зобе и раке щитовидной железы.

Важно отметить, что метод иммунофлюоресцентной гибридизации in situ (FISH — fluorescence in situ hybridization) позволяет выявлять не только мужские клетки среди женских, но и женские среди мужских, что дает возможность использовать методику для обнаружения и фетального, и материнского МХ. Для обнаружения материнского МХ также может применяться HLA-типирование на основе ПЦР с выявлением не унаследованных антигенов главного комплекса гистосовместимости [33]. Бесспорным преимуществом FISH является возможность точного подсчета микрохимерных клеток [34]. Следует также учитывать различную чувствительность используемых методик. С помощью количественной ПЦР одна фетальная клетка может быть обнаружена среди 100 000 [29], с помощью FISH — среди 2 000 000 материнских [35].

Так, Lepez T. и соавт. проводили исследование, целью которого было выявление и описание фетальных клеток в крови женщин в послеродовом периоде с аутоиммунными заболеваниями щитовидной железы (ЩЖ) и без. Для подсчета количества клеток мужского плода использовали флуоресцентную гибридизацию in situ (FISH) и повторную FISH [36].

Мононуклеарные клетки периферической крови (МКПК) были выделены из образцов крови пациента с этилендиаминтетрауксусной кислотой (ЭДТА), методом центрифугирования в градиенте плотности на Ficoll-Paque Plus (GE Healthcare, Diegem, Бельгия) в соответствии с инструкциями производителя. Из каждого образца 1 000 000 МКПК были помещены в цитоцентрифугу на 4 предметных стекла. Предметные стекла были высушены на воздухе и зафиксированы в течение 5 минут в фиксаторе Карнуа (3:1 метанол (Fisher scientific, Лестершир, Великобритания): уксусная кислота (Sigma-Aldrich, Bornem, Бельгия)). Клетки мужского плода отличались от клеток матери c помощью метода FISH с использованием ДНК-зондов CEP X SpectrumOrange/CEP Y SpectrumGreen (Vysis, Abbott Molecular, Иллинойс, США). Клетки мужского плода показали одну точку SpectrumGreen Y-FISH и одну точку SpectrumOrange X-FISH, тогда как материнские клетки показали две точки SpectrumOrange X-FISH (рис. 1).

**Figure:**
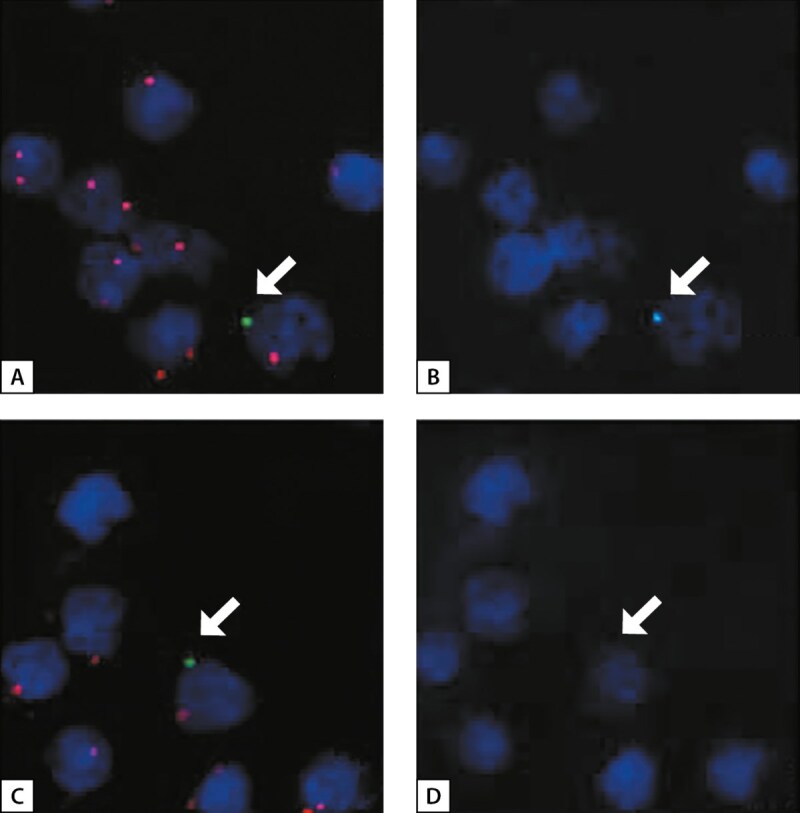
Рисунок 1. FISH и повторный FISH. A. FISH женских клеток, показывающий два пятна SpectrumOrange X- FISH и предполагаемой мужской клетки, указанной стрелкой, показывающий одно пятно SpectrumOrange X-FISH и одно пятно SpectrumGreen Y-FISH.B. Повторный FISH женских клеток и предполагаемой мужской клетки, показывающий отсутствие пятен SpectrumAqua Y-FISH в женских клетках. Напротив, мужская клетка показывает один сигнал SpectrumAqua Y-FISH в том же самом месте, что и пятно SpectrumGreen Y-FISH на изображении A (указано стрелкой), что указывает на то, что это настоящая мужская клетка.C. FISH женских клеток и одной предполагаемой мужской клетки, указанной стрелкой.D. Повторный FISH мужской клетки (C) не показывает пятно SpectrumAqua Y FISH. Пятно SpectrumGreen Y FISH, вероятно, было вызвано клеточным детритом или частицами пыли. Пятно SpectrumOrange X FISH этой клетки больше других пятен SpectrumOrange, что может указывать на два пятна SpectrumOrange X FISH, лежащих очень близко друг к другу (адапт. из [36] Lepez T. и соавт.).

У всех пациентов с аутоиммунными заболеваниями щитовидной железы, включенных в данное исследование, в крови были обнаружены ФМК. Наибольшее количество фетальных клеток наблюдалось в несортированной фракции МКПК пациентов с болезнью Грейвса (от 14 до 29 фетальных клеток на миллион материнских клеток), за которыми следует тиреоидит Хашимото (от 7 до 11), по сравнению с низким количеством фетальных клеток, обнаруженных у здоровых добровольцев (от 0 до 5). Это указывает на более высокую степень микрохимеризма при аутоиммунных заболеваниях щитовидной железы по сравнению со здоровыми лицами. Более того, значительно больше фетальных клеток было обнаружено у пациентов с болезнью Грейвса по сравнению с пациентами с тиреоидитом Хашимото (p=0,0061) [36].

## Аутоиммунные заболевания щитовидной железы (АЗЩЖ)

Беременность и послеродовой период оказывают сильное влияние на развитие аутоиммунных заболеваний щитовидной железы. Так, течение болезни Грейвса, как правило, улучшается во время беременности, а рецидив происходит уже только после родов, тогда как послеродовой тиреоидит вызывается деструктивным аутоиммунным процессом в течение первых нескольких месяцев после родов. ФМК широко изучались при аутоиммунных заболеваниях щитовидной железы [37].

Существует ряд гипотез о возможных механизмах, с помощью которых ФМК плода могут влиять на аутоиммунный статус матери. Во-первых, неблагоприятное влияние фетальных клеток может заключаться в способности вызывать аутоиммунные заболевания щитовидной железы, инициируя реакцию «трансплантат против хозяина», или сам материнский организм может инициировать реакцию «хозяин против трансплантата». Во-вторых, фетальные клетки могут оказывать положительное влияние, участвуя в регенерации тканей материнского организма. В-третьих, фетальные клетки могут просто присутствовать в щитовидной железе как свидетели, не оказывая при этом никакого влияния на аутоиммунитет.

Гипотеза 1. Во время беременности циркулирующие фетальные клетки не вызывают заболеваний, что связано с явлением иммунологической толерантности при беременности. Однако же в послеродовом периоде, когда плацентарный иммунитет утрачивается, ФМК могут активироваться из-за различных факторов: триггерами могут быть вирусные или бактериальные агенты, лекарственные препараты или аномальные тканевые белки [20]. Эти активированные ФМК инициируют местный иммунный ответ. Они могут действовать как эффекторные клетки, инициируя гипотезу: МХ вызывает реакцию «трансплантат против хозяина» Считается, что активация незрелых Т-клеток плода, моноцитов, макрофагов и NK-клеток, а также продукция воспалительных цитокинов и хемокинов инициируют аутоиммунное заболевание [24][28].

Гипотеза 2. Эта гипотеза заключается в том, что фетальные клетки могут быть мишенью: МХ вызывает реакцию «хозяин против трансплантата»: они могут быть признаны частично аллоиммунными и вызывать аутоиммунную реакцию путем прямого ответа материнских клеток на фетальные клетки или путем молекулярной мимикрии между фетальными антигенами и интратироидными материнскими антигенами [38–40]. После родов, когда плацентарное иммунное подавление теряется, фетальные иммунные клетки могут активироваться и инициировать аутоиммунную реакцию, основанную на несоответствиях HLA.

В случае прямого ответа на микрохимерные клетки ФМК могут инициировать РТПХ против материнских антигенов, при котором активируются интратиреоидные материнские аутореактивные Т-клетки, что в итоге приводит к тому, что материнские клетки вызывают повреждение ткани. В другом же случае фетальные антигенпрезентирующие клетки представляют материнские антигены иммунным клеткам, что приводит к иммунной реакции матери против ее собственных клеток [41].

При молекулярной мимикрии материнские клетки начинают иммунную реакцию против фетальных антигенов, но из-за сходства между фетальными антигенами и материнскими тиреоидными аутоантигенами возникает аутоиммунитет к щитовидной железе [41]. Данные механизмы проиллюстрированы на рисунке 2.

**Figure:**
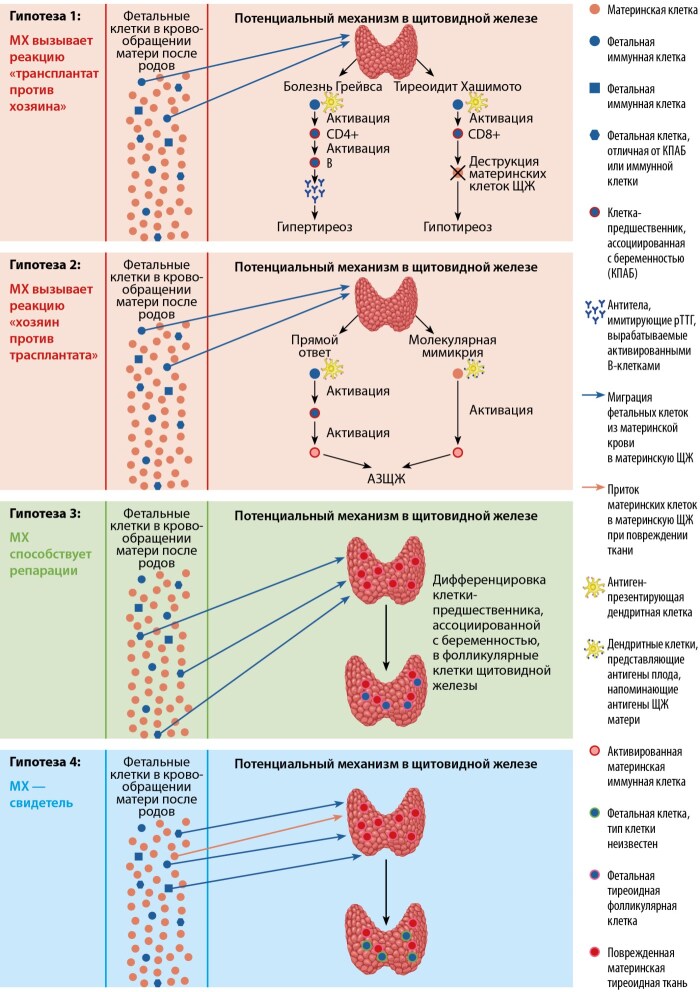
Рисунок 2. Потенциальные механизмы вредного (красный), полезного (зеленый) и безвредного (синий) микрохимеризма в щитовидной железе (адапт. из [20] Lepez T. и соавт.).

Гипотеза о том, что ФМК могут играть роль в патогенезе АЗЩЖ, была дополнительно поддержана обнаружением значительно более высокой степени микрохимерных клеток в щитовидной железе женщин с тиреоидитом Хашимото и болезнью Грейвса по сравнению с женщинами без аутоиммунных заболеваний щитовидной железы [42].

## Фетальный микрохимеризм и болезнь Грейвса

Согласно исследованию Jansson и соавт., у 2 из 3 женщин, у которых развивается болезнь Грейвса, заболевание начинается после родов, что предполагает важную роль иммуномодулирующих событий после родов. Наличие ФМК в материнской ЩЖ, которые могут активироваться в послеродовой период, когда материнская иммунная супрессия угасает, может указывать на важную роль фетальных клеток в патогенезе аутоиммунных заболеваний ЩЖ [20][5].

Как правило, клиническое течение болезни Грейвса улучшается по мере развития беременности, параллельно снижению уровня аутоантител к рецептору тиреотропного гормона (рТТГ). Отражением этого клинического улучшения может быть не только количество концентрации антител к рТТГ, но и качество (биологическое действие) этих антител, которое может смещаться в пользу блокирующих антител [43], хотя не все эксперты разделяют это мнение [44]. Неустойчивое клиническое течение болезни Грейвса является распространенной находкой во время беременности с обострением в течение первых трех месяцев и улучшением в последнем триместре [45]. В послеродовом периоде, когда исчезает связанное с беременностью иммунно-привилегированное состояние, может возникнуть рецидив и обострение болезни Грейвса. Это обычно происходит через 4–12 месяцев после родов [46]. Эпидемиологические исследования показывают, что около 60% женщин детородного возраста заболевают болезнью Грейвса в течение первого года после родов [47][48], тогда как частота рецидивов варьируется от 30 до 70% случаев [49]. Повышенный риск развития болезни Грейвса после беременности может быть выше у взрослых пациентов (>35 лет), который сохраняется в течение нескольких лет после родов [29][50].

По данным исследования Yan Z. и соавт. [33], временной интервал между беременностью и началом болезни Грейвса составляет 1 год в 60% случаев. На сегодняшний день нет данных, объясняющих может ли эта временная последовательность быть связана с наличием активированных интратироидных фетальных клеток или быть следствием других факторов, кроме фетального микрохимеризма. Однако наличие остаточных фетальных клеток в материнской щитовидной железе, которые активируются в послеродовом периоде по мере потери материнской иммуносупрессии, остается привлекательным объяснением послеродовой манифестации аутоиммунных тиреопатий [29].

## Фетальный микрохимеризм и аутоиммунный тиреоидит

Аутоиммунный тиреоидит (АИТ) имеет выраженную женскую предрасположенность, соотношение женщин и мужчин составляет 10:1. Более того, у женщин это заболевание, как правило, чаще встречается в возрасте от 30 до 50 лет и часто выявляется в первые годы после родов [47][51][52].

В нескольких клинических исследованиях были обнаружены мужские клетки в образцах щитовидной железы женщин, которым ранее был поставлен диагноз «тиреоидит Хашимото». Наличие чужеродных клеток в аутоиммунных пораженных щитовидных железах варьировалось от 38 до 83%. Процентные различия между тиреоидитом Хашимото и контрольными группами в отдельных исследованиях являются постоянными.

Srivatsa и соавт. выполнили анализ флуоресцентной гибридизации in situ (FISH) для хромосом X и Y на залитой парафином ткани щитовидной железы 29 женщин, перенесших тиреоидэктомию, а также на восьми нормальных щитовидных железах [32]. Авторы сравнили наличие мужских клеток, предположительно фетального происхождения, а также изучили истории беременности и истории болезни женщин. В результате исследования были обнаружены мужские клетки у 12 из 20 пациенток с известным анамнезом детей мужского пола и у четырех из девяти пациенток без известного анамнеза детей мужского пола. Из женщин с детьми мужского пола мужские клетки присутствовали чаще всего в щитовидной железе женщин с тиреоидитом Хашимото (83%), и несколько реже в щитовидной железе женщин с другими, невоспалительными заболеваниями [53].

Гипотеза 3. Некоторые данные свидетельствуют о том, что фетальные клетки-предшественники способны дифференцироваться в зрелые клетки, специфичные для определенной ткани, в травмированных материнских органах. Разнообразие типов клеток, в которые могут дифференцироваться микрохимерные клетки, свидетельствует о том, что в этом участвует очень ранний тип стволовых клеток — так называемые «клетки-предшественники, ассоциированные с беременностью» (КПАБ). Таким образом, наличие микрохимерных клеток-предшественников во взрослой щитовидной железе может быть потенциальным источником регенерации ткани.

Аргументом в пользу положительного влияния микрохимерных клеток является тот факт, что ФМК также были обнаружены у здоровых женщин без признаков аутоиммунных заболеваний. Протективная роль микрохимеризма в основном была описана в случаях восстановления тканей при раке [54].

Гипотеза 4. Данная гипотеза предполагает, что фетальные микрохимерные клетки являются «свидетелями» и не участвуют в возникновении или обострении АИТ [55]. Возможно, что микрохимерные клетки равномерно распределены по всему телу. Если происходит повреждение ткани, фетальные клетки будут привлечены из-за воспалительных инфильтратов, в связи с чем уровень микрохимеризма в пораженной ткани будет выше по сравнению со здоровой тканью, а это означает, что нет никакой связи с патогенезом самого заболевания. Фетальные интратироидные клетки, даже иммунные клетки, могут быть отражением продолжающейся местной иммунной реакции без активного участия фетальных микрохимерных клеток. Из-за повреждения кровеносных сосудов фетальные клетки могут проникать в поврежденную ткань, не играя активной роли в повреждении или восстановлении ткани.

Аргументом в пользу этой гипотезы является тот факт, что три крупных эпидемиологических исследованиях не смогли продемонстрировать связь между беременностью, паритетом (количество предшествующих родов), абортом и наличием аутоантител к щитовидной железе или дисфункцией щитовидной железы. Клетки плода были лишь следствием беременности. Напротив, одно исследование случай-контроль указало на паритет как на потенциальный фактор риска аутоиммунных заболеваний щитовидной железы, показав более высокие уровни аутоантител к щитовидной железе у женщин с предыдущими беременностями по сравнению с нерожавшими женщинами. Однако совместимость HLA между клетками плода и матери может быть более важным фактором риска, чем количество беременностей в инициации аутоиммунной реакции микрохимерными клетками плода [1].

## Сахарный диабет 1 типа

Сахарный диабет 1 типа (СД1) — это аутоиммунное заболевание, при котором бета-клетки, продуцирующие инсулин, становятся мишенью для иммунной системы. Основным известным генетическим фактором риска для СД1 является генотип человеческого лейкоцитарного антигена (HLA). Факторы окружающей среды недостаточно изучены, но выдвинута гипотеза, что пренатальные и ранние факторы окружающей среды влияют на риск СД1. При этом сахарный диабет 1 типа встречается примерно в два раза чаще у потомства мужчин с СД1, чем у потомства женщин с СД1, но причины этой разницы неясны [56].

Как сообщалось выше, исследования АЗЩЖ были сосредоточены на ФМК, тогда как СД1 в основном изучался в контексте с микрохимеризмом материнских клеток (ММК). В частности, с использованием панели количественных анализов ПЦР в реальном времени, нацеленных на ненаследственные материнские антигены, было обнаружено, что распространенность циркулирующих материнских клеток была значительно выше у пациентов с СД1 (51%), чем у здоровых братьев и сестер (33%) и у неродственных здоровых субъектов (17%). Повышенные уровни ММК у пациентов с СД1 не были связаны с гаплотипами восприимчивости DQB1*0201-DRB1*03 и DQB1*0302-DRB1*04, таким образом исключая, что материнские клетки могли быть источником генотипов восприимчивости HLA. Было отмечено, что у пациентов, унаследовавших от матери гаплотип DQB1*0302-DRB1*04, ассоциированный с СД1, ММК встречался чаще, чем у тех, кто унаследовал данный гаплотип от отца (70 против 14%). Не было обнаружено корреляций между уровнями микрохимерных клеток и полом, возрастом и временем от начала заболевания [57].

Более подробную информацию о возможной роли ММК в развитии СД1 удалось получить с помощью FISH и конфокальной визуализации, при изучении ткани поджелудочной железы у пациентов мужского пола и здоровых лиц. Было обнаружено, что материнские клетки располагаются небольшими группами, кластерами вблизи или внутри островков поджелудочной железы, что предполагает их активную репликацию, также было обнаружено, что они вырабатывают инсулин. Материнские (женские) островковые β-клетки составили 0,39–0,96% от общего числа островковых β-клеток у пациентов с СД1, тогда как в поджелудочной железе пациентов без СД1 они встречались крайне редко. Точная роль женских островковых β-клеток у пациентов мужского пола с СД1 пока не выяснена, но выдвинута гипотеза, что они могут быть целями для аутоиммунитета, в этом случае роль ММК будет пагубной [57][58].

Так, Vanzyl и соавт. в своем исследовании изучали образцы поджелудочной железы, которые были взяты от шести мужчин с СД1 и четырех мужчин из контрольной группы. Для обнаружения X и Y-хромосом использовались флуоресцентно-меченые зонды. Не менее 1000 клеток, обычно 4000–8000 клеток, прошли конфокальную визуализацию для каждой поджелудочной железы. Частота МКК была выше в поджелудочных железах больных СД1 (диапазон 0,31–0,80%, среднее значение 0,58%), чем в контрольной группе (0,24–0,50%, среднее значение 0,38%, p=0,05). Кластеры из 2–3 ММК иногда обнаруживались в поджелудочных железах, особенно в поджелудочных железах больных СД1, что предполагает репликацию этих клеток. Для фенотипирования клеток материнского происхождения были проведены сопутствующая FISH и иммунофлуоресцентное окрашивание на инсулин или CD45. Были идентифицированы инсулин-положительные и инсулин-отрицательные ММК, что указывает на то, что ММК вносит вклад в экзокринные и эндокринные компартменты. CD45-положительных ММК не наблюдалось. Эти данные подтверждают наличие материнских клеток в поджелудочной железе человека и предыдущие наблюдения о том, что уровни ММК выше в поджелудочной железе больных СД1 по сравнению с контрольной группой. ММК, по-видимому, не являются иммунными эффекторными клетками. И те клетки, которые окрашиваются положительно на инсулин в неповрежденных островках в ткани СД1, являются здоровыми, без доказательств того, что они являются фокусом иммунной атаки. Это исследование подтверждает гипотезу о том, что материнские стволовые клетки способны пересекать плацентарный барьер и дифференцироваться как в эндокринные, так и в экзокринные клетки, но требуется более подробная характеристика ММК в поджелудочной железе [58].

Tapia G. et al. разработали чувствительные аллель-специфические droplet digital polymerase chain reaction (ddPCR)-анализы, чтобы проверить, имеют ли ММК пуповинной крови какую-либо прогностическую ценность для СД1 у детей, но не обнаружили значительной связи. Участники норвежского когортного исследования матери и ребенка были типированы по HLA класса II для определения ненаследуемых материнских антигенов (НМА). Были разработаны и проверены капельные цифровые (droplet digital) полимеразные цепные реакции, специфичные для общего HLA класса II НМА (HLADQB1*03:01, *04:02 и *06:02/03). ММК оценивались как количество материнской ДНК в фетальном кровообращении с помощью специфической НМА ddPCR, измеренной в ДНК пуповинной крови 71 ребенка, у которых позже развился СД1. А также 126 контрольных лиц в пределах когорты. В результате были обнаружены определяемые количества ММК в 34 из 71 будущих случаях СД1 (48%) и 53 из 126 контрольных случаев (42%) (скорректированное отношение шансов [aOR] 1,27, 95% доверительный интервал (CI) 0,68–2,36), и не обнаружено значительной разницы в рангах количеств ММК между случаями и контрольными случаями (Mann-Whitney P=46). Была ассоциация в подгруппе ненаследуемых материнских антигенов HLA-DQB1*03:01 с более поздним дебютом СД1 (aOR 3,89, 95%CI 1,05–14,4). ММК в пуповинной крови не был значимо связан с ММК при диагностике СД1. Таким образом, данные результаты не подтвердили гипотезу о том, что высокий уровень ММК в пуповинной крови предсказывает риск СД1 [53].

Обращает на себя внимание исследование, проведенное Fjeldstad с соавт., в которое было включено 122 беременные женщины (прегестационный СД, n=77, гестационный СД (ГСД), n=45). Образцы крови матери и плода были генотипированы по различным локусам человеческого лейкоцитарного антигена (HLA) и другим полиморфизмам для выявления аллелей, специфичных для плода. Использовались анализы ПЦР для количественной оценки ФМК в лейкоцитарном слое периферической крови матери. Отрицательная биномиальная регрессия с поправкой на вмешивающиеся факторы применялась для оценки количества ФМК. При прегестационной форме СД увеличение циркулирующего ФМК коррелировало с повышением гликированного гемоглобина (≥6,0 %) (коэффициент частоты обнаружения (DRR)=4,9, p=0,010) и повышением на 1000 пг/мл антиангиогенного биомаркера растворимой fms-подобной тирозинкиназы-1 (sFlt-1) (DRR=1,1, p=0,011). При ГСД повышенный уровень ФМК коррелировал с повышенными результатами 2-часового перорального теста на толерантность к глюкозе (DRR=2,3, p=0,046) и массой тела при рождении <10‑го или >90‑го процентиля (DRR=4,2, p=0,049). Эти результаты подтверждают новую гипотезу ученых о том, что ФМК коррелирует с плохим контролем глюкозы и различными аспектами плацентарной дисфункции при СД. Способствует ли повышенный уровень ФМК при беременностях с плохим контролем глюкозы, с плацентарной дисфункцией, риску преэклампсии (при диабетической беременности) и повышенному риску хронических сердечно-сосудистых заболеваний в более позднем возрасте, еще предстоит изучить [59].

Интересны результаты исследования, проведенного японскими исследователями Ushijima и соавт., целью которого было выяснить распространенность и степень выраженности материнского микрохимеризма у японских детей с СД1, а также его влияние на фенотипическую изменчивость. Изучили 153 японских ребенка с СД1, включая 124 ребенка с положительным результатом на аутоантитела к β-клеткам, и их 71 здорового брата или сестру. Количество циркулирующих микрохимерных клеток на 10⁵ клеток хозяина оценивалось с помощью количественной ПЦР, нацеленной на непередаваемые аллели материнского лейкоцитарного антигена человека. Результаты сравнивались с предыдущими данными по белым европейцам. Фенотипическое сравнение проводилось между носителями материнского микрохимеризма и неносителями с диабетом. Материнский микрохимеризм был обнаружен у 15% детей с диабетом 1 типа с положительным результатом на аутоантитела, у 28% детей с диабетом 1 типа с отрицательным результатом на аутоантитела и у 16% здоровых братьев и сестер. Не было выявлено никаких различий в распространенности или уровнях материнского микрохимеризма среди трех групп или между детьми с диабетом 1 типа и их здоровыми братьями и сестрами. Более того, носители и неносители материнского микрохимеризма продемонстрировали схожие фенотипы [60].

## Заключение

Рассмотренные в данном обзоре литературные данные указывают на то, что микрохимеризм является достаточно интересным, актуальным и новым феноменом. Безусловно, имеется определенная взаимосвязь между обнаружением микрохимерных клеток и развитием таких эндокринных патологий, как аутоиммунные заболевания щитовидной железы и сахарный диабет 1 типа. На данный момент существуют различные гипотезы о возможных механизмах влияния микрохимеризма на формирование эндокринных заболеваний. Однако точный вклад этого явления в развитие данных патологий неоднозначен и требует дальнейшего рассмотрения, изучения и проведения более расширенных и углубленных исследований.

## Дополнительная информация

Источники финансирования. Работа выполнена по инициативе авторов без привлечения финансирования.

Конфликт интересов. Авторы декларируют отсутствие явных и потенциальных конфликтов интересов, связанных с содержанием настоящей статьи.

Участие авторов. Все авторы одобрили финальную версию статьи перед публикацией, выразили согласие нести ответственность за все аспекты работы, подразумевающую надлежащее изучение и решение вопросов, связанных с точностью или добросовестностью любой части работы.
